# Successful catheter ablation in an octogenarian with persistent atrial fibrillation complicated by cor triatriatum sinister: a case report

**DOI:** 10.1093/ehjcr/ytae490

**Published:** 2024-09-10

**Authors:** Yusuke Okuyama, Atsushi Tamura, Kohei Ueda, Shunzo Matsuoka, Yoshihisa Nakagawa

**Affiliations:** Department of Cardiovascular Medicine, Uji-Tokushukai Medical Center, 145 Ishibashi, Makishima-cho, Uji, Kyoto Prefecture, 611-0041, Japan; Department of Cardiovascular Medicine, Uji-Tokushukai Medical Center, 145 Ishibashi, Makishima-cho, Uji, Kyoto Prefecture, 611-0041, Japan; Department of Clinical Engineering, Uji-Tokushukai Medical Center, Uji, Kyoto Prefecture, 611-0041, Japan; Department of Cardiovascular Medicine, Uji-Tokushukai Medical Center, 145 Ishibashi, Makishima-cho, Uji, Kyoto Prefecture, 611-0041, Japan; Department of Cardiovascular Medicine, Shiga University of Medical Science, Otsu, Shiga Prefecture, 520-2192, Japan

**Keywords:** Cor triatriatum sinister, Catheter ablation, Atrial fibrillation, Octogenarian, Preoperative assessment, Case report

## Abstract

**Background:**

Cor triatriatum sinister (CTS) is a rare congenital heart defect sometimes complicated with atrial fibrillation (AF). Catheter ablation (CA) relieves the AF-associated symptoms, but CA for AF with CTS has been reported rarely. Because CTS can be associated with other congenital heart disease, detailed preoperative assessment is important.

**Case summary:**

An 80-year-old man was referred to our institution for shortness of breath that had persisted for 2 months when he was first diagnosed with AF. Transthoracic echocardiography revealed an enlarged left atrium (LA) divided into two chambers by a membrane. Transoesophageal echocardiography showed the membrane extending from the fossa ovalis (FO) to the Coumadin ridge, with the accessory (dorsal) chamber (AC) in closer proximity to the FO. Computed tomography showed that all pulmonary veins (PVs) flowed into the AC, with no PV anomalies. No other heart anomaly was identified, with no thrombus in the LA. With these findings, PV isolation (PVI) with CA was considered safe. Transseptal puncture was performed with intracardiac echocardiography for precise catheterization of the AC. Pulmonary vein isolation was performed successfully. The patient was discharged 4 days after the procedure, without any complications. His symptoms improved post-procedure, and sinus rhythm was maintained without antiarrhythmic drug therapy during the 18-month follow-up.

**Discussion:**

Cor triatriatum sinister is a rare anomaly that accounts for 0.1% of all congenital heart diseases. Cor triatriatum sinister sometimes complicated with symptomatic AF. Detailed preoperative anatomical assessment with multiple imaging modalities helped us achieve safe and effective CA for a patient with AF and CTS, even in an octogenarian.

**Learning points**

- Safe and effective pulmonary vein isolation in patients with cor triatriatum sinister (CTS) is feasible even in an octogenarian.
- Recognizing anatomical features in the atria is very important for safe and effective catheter ablation in patients with congenital heart diseases, such as cor triatriatum, sinister because the feasibility of treatment depends on anatomical complexity.
- Detailed multi-modality preoperative and intraoperative investigation to identify the in specific anatomy helped us perform a successful procedure in a patient with CTS complicated with atrial fibrillation.

## Introduction

Cor triatriatum sinister (CTS) is a rare anomaly that accounts for 0.1% of congenital heart diseases.^1^ In CTS, a fibromuscular membrane divides the left atrium (LA) into two chambers,^2,3^ and the condition is sometimes associated with atrial fibrillation (AF) and heart failure.^4^ Approximately 30% of CTS cases are complicated with AF;^4^ however, catheter ablation (CA) for AF with CTS has rarely been reported. Cor triatriatum sinister can be associated with other congenital heart disease such as atrial septal defects, pulmonary vein (PV) malformations, and abnormal PV return.^5^ Therefore, detailed preoperative assessment is necessary to perform CA in patients with CTS. However, few reports have discussed the importance of the preoperative assessment for a safe and effective CA in these patients. Here, we report a patient with symptomatic persistent AF complicated by CTS who was first diagnosed at an age of 80 years. This patient with CTS underwent a detailed preoperative assessment with transthoracic echocardiography (TTE), transoesophageal echocardiography (TEE), multidetector computed tomography (MDCT), and intraoperative intracardiac echocardiography (ICE) to accomplish a safe and effective CA, even as an octogenarian with CTS.

## Summary figure

**Figure:**
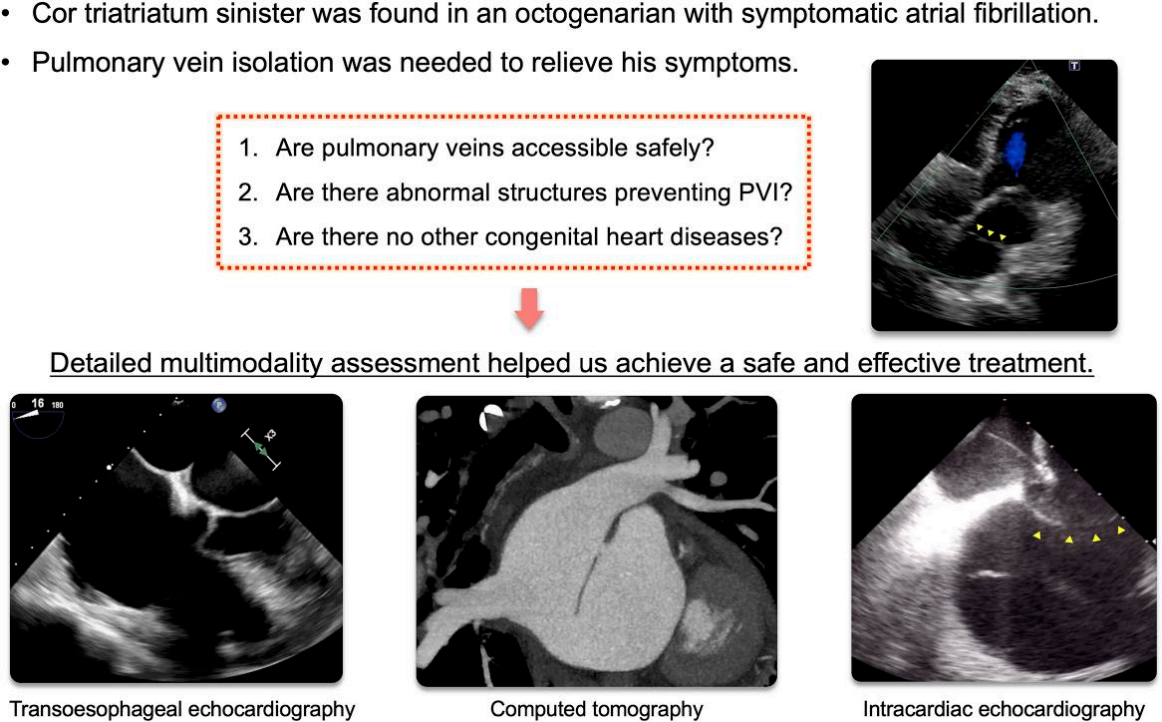


## Case presentation

**Figure 1:**
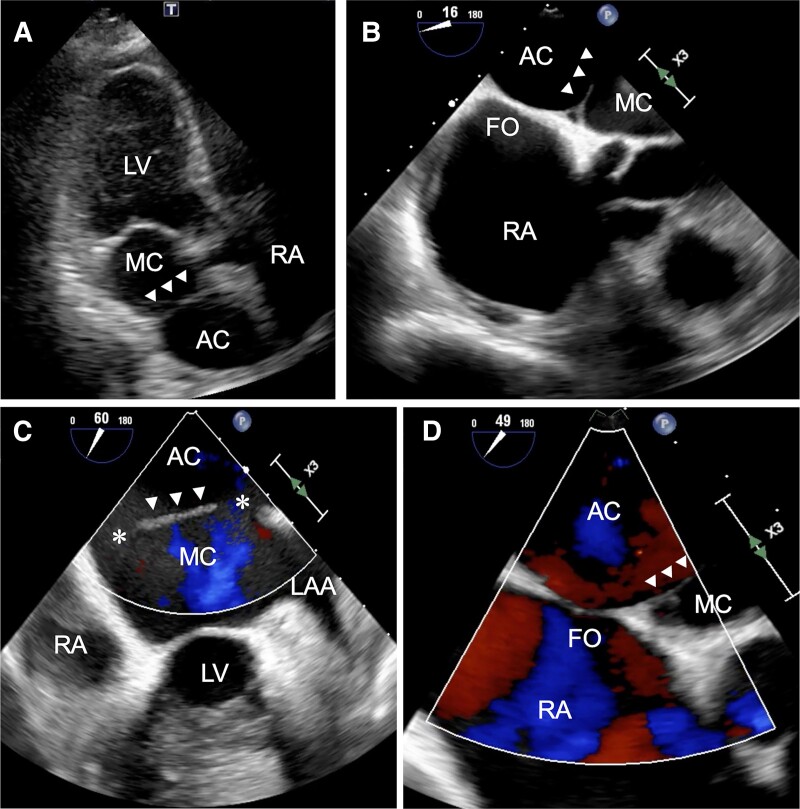
Echocardiography findings. (*A*) Transthoracic echocardiography. (*B* and *C*) Transoesophageal echocardiography: the left atrium is divided into two chambers by a membrane. (*D*) Transoesophageal echocardiography: the membrane has two orifices (*). LAA, left atrial appendage; RA, right atrium; LV, left ventricle; FO, fossa ovalis; AC, accessory chamber; MC, main chamber; TTE, transthoracic echocardiography; TEE, transoesophageal echocardiography. Arrow heads, membrane.

**Figure 2:**
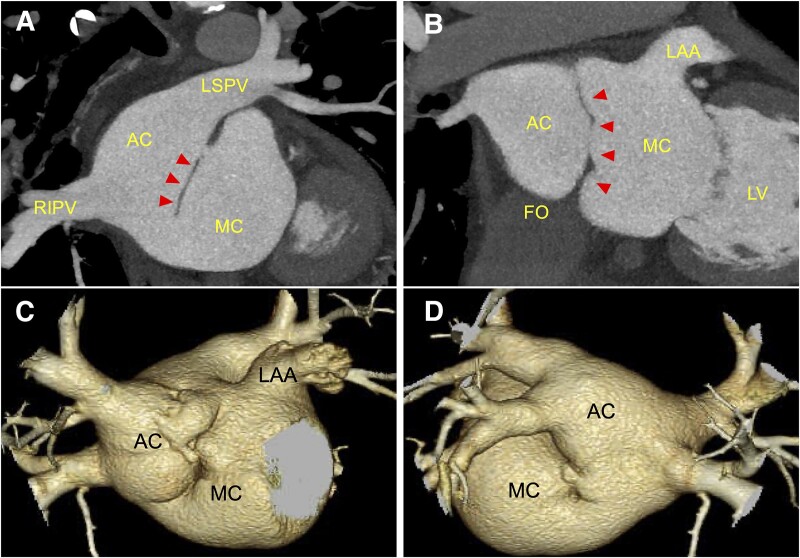
Multidetector computed tomography findings. (*A* and *B*) Coronal sections. The fossa ovalis is in contact with the accessory chamber. (*C* and *D*) Three-dimensional reconstruction. All pulmonary veins flow into the accessory chamber. LAA, left atrial appendage; LV, left ventricle; FO, fossa ovalis; AC, accessory chamber; MC, main chamber; RIPV, right inferior pulmonary vein; LSPV, left superior pulmonary vein. Arrow heads, membrane.

An 80-year-old man was referred to our institution for shortness of breath of 2 months’ duration. He had been receiving therapy for hypertension with a calcium channel blocker at a local clinic. He was first diagnosed with AF on an electrocardiography at the local clinic 2 months earlier. Physical examination revealed no oedema and oxygen saturation within the normal range. On auscultation, systolic murmur with an irregular rhythm were heard. Electrocardiography recorded AF when he visited our institution, and we diagnosed persistent AF. Transthoracic echocardiography revealed an enlarged left atrium (LA) divided into two chambers by a membrane (*Figure 1A*). The LA diameter was 43 mm, the left ventricular ejection fraction was 64%, and mild mitral and tricuspid regurgitation were observed. The tricuspid regurgitation maximum pressure gradient was 23 mmHg. Transoesophageal echocardiography demonstrated a membrane extending from the fossa ovalis (FO) to the Coumadin ridge that divided the LA into two chambers: dorsal [accessory (AC)] and ventral (main) chambers (*Figure 1B* and *C*). This membrane had an orifice on both the atrial septal and left auricular sides, with blood flow from the AC to the main chamber (*Figure 1C*). The atrial septal attachment of the membrane was deviated towards the main chamber, and the AC was in greater contact with the FO, comparatively (*Figure 1B* and *D*). All PVs flowed into the AC, with no visible atrial septal defects. Multidetector computed tomography also showed that all PVs returned to the AC, with no PV anomalies. Each orifice measured 10 mm in diameter. The majority of the FO had contacted the AC (*Figure 2*). No other congenital heart anomalies were identified, and there was no thrombus in the LA.

Transoesophageal echocardiography and MDCT findings confirmed that the FO had sufficient area for transseptal puncture and that transseptal puncture could reach the AC. Therefore, we considered that PV isolation (PVI) with CA could be performed safely.

**Figure 3:**
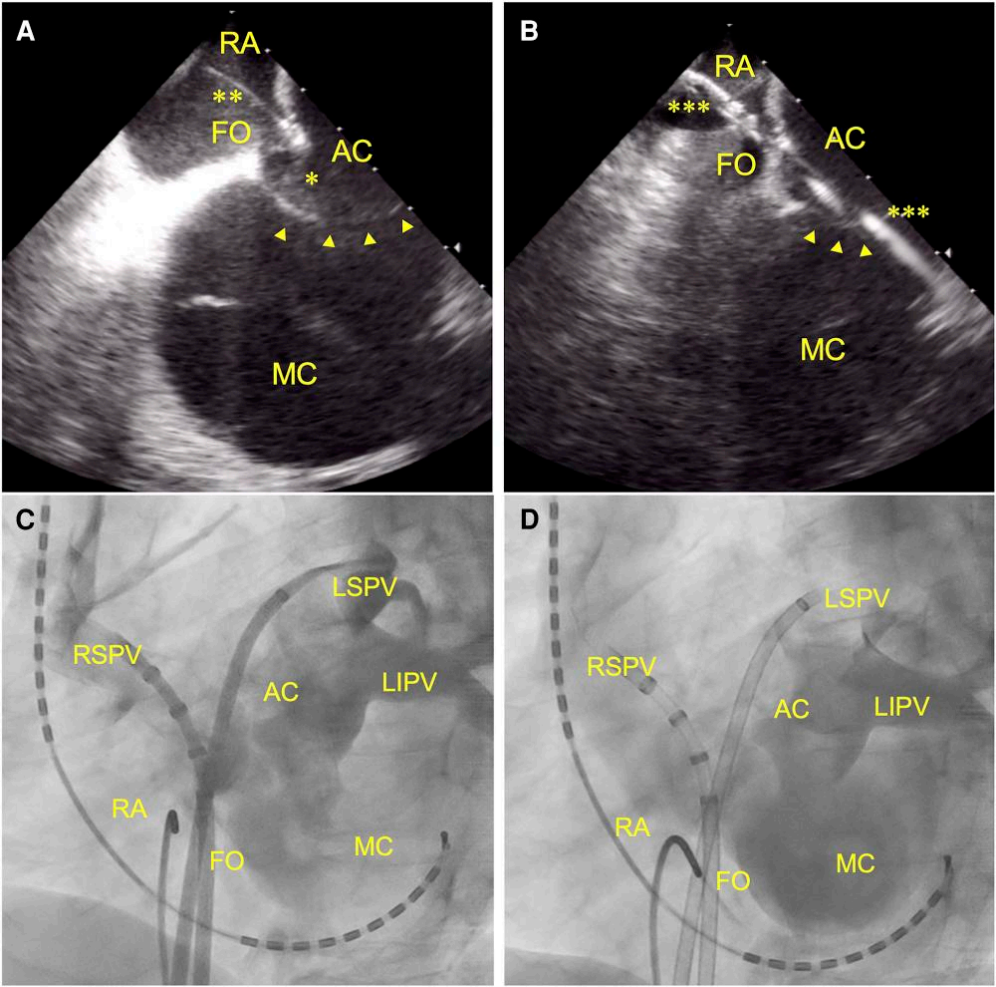
Transseptal puncture with intracardiac echocardiography (*A* and *B*) and pulmonary venography (*C* and *D*). (*A*) Before transseptal puncture using a transseptal needle (**). The needle tip (*) is pushing the fossa ovalis towards the accessory chamber. (*B*) After transseptal puncture. Guidewires (***) are inserted into the accessory chamber through the fossa ovalis. (*C* and *D*) Confirmation of precise catheterization into the accessory chamber with contrast infusion. AC, accessory chamber; MC, main chamber; FO, fossa ovalis; RA, right atrium; RSPV, right superior pulmonary vein; LSPV, left superior pulmonary vein; LIPV, left inferior pulmonary vein. Arrow heads, membrane.

After obtaining written informed consent from the patient, CA for symptomatic persistent AF was performed. We performed transseptal puncture with ICE (SOUNDSTAR; Biosense Webster, Diamond Bar, CA, USA) to place the catheter precisely in the AC and avoid accidental needle puncture of the membrane (*Figure 3A* and *B*). Two transseptal sheaths were inserted from the same site. Contrast injection into the AC through these sheaths confirmed successful catheterization into the AC. Contrast infusion clarified anastomosis between the main chamber and AC as well as the anatomical locations of all PVs and the AC (*Figure 3C* and *D*).

**Figure 4:**
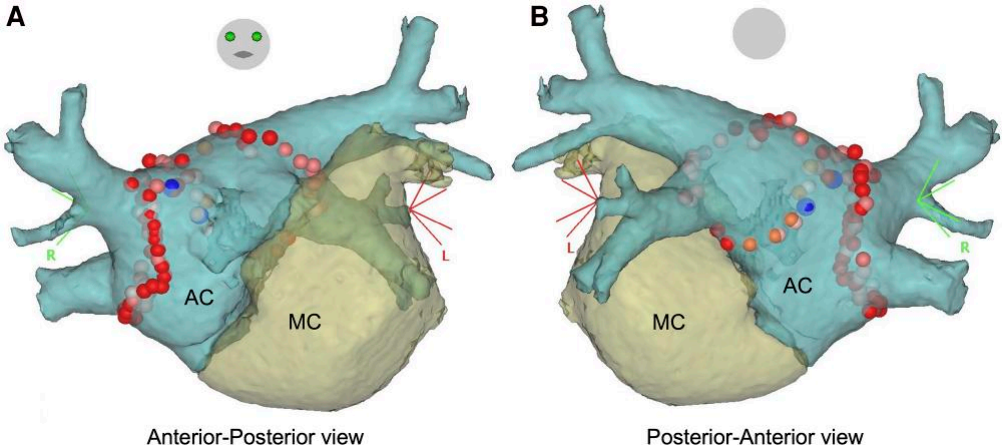
Pulmonary vein isolation with three-dimensional images in an electroanatomical mapping system. Images were reconstructed from multidetector computed tomography information. All pulmonary veins flowed into the accessory chamber. The dots show the ablation points. AC, accessory chamber; MC, main chamber.

A contact force-sensing irrigated ablation catheter (Navistar ThermoCool SmartTouch, Biosense Webster) and a circular 20-pole catheter (Lasso, Biosense Webster) were inserted into the AC via the transseptal sheaths. We reconstructed three-dimensional shells of the LA (main chamber and AC) and PVs using data from MDCT and a merged electroanatomical mapping system (CARTO system; Biosense Webster). In the shells, we used a different colour for each chamber to distinguish the chambers and easily visualize the catheters’ locations (*Figure 4*). After sinus rhythm was restored by electrical cardioversion, PVI was performed in the antrums of the right and left PVs. The PVI endpoint was confirmation of bidirectional conduction blocks between the PVs and the AC. First-pass isolation was achieved on both sides of the PVs. After PVI, superior vena cava isolation and linear ablation for the cavotricuspid isthmus were performed. The patient was discharged 4 days after the procedure, without complications. His symptoms improved post-procedure, and sinus rhythm was maintained without antiarrhythmic drug therapy during the 18-month follow-up.

## Discussion

Two points are important in this case. First, this was an elderly patient with persistent AF complicated by CTS, which was first diagnosed at an age of 80 years. Catheter ablation was required for the AF to improve his symptoms. Second, detailed anatomical characterization of the LA and PVs in this patient with CTS using imaging modalities before and during the procedure helped achieve safe and effective treatment.

Cor triatriatum sinister is diagnosed at a median age of 43 (interquartile range: 30–60) years.^4^ Embryologically, CTS is thought to result from incomplete resorption of the common PV and, thus, a membrane remains left in the LA.^2^ Symptoms depend on the size of the window in the membrane.^6^ Sometimes, severe obstruction of mitral inflow due to a small window results in pulmonary congestion, and surgical resection of the membrane is needed.^6^ The wide window in our case might have delayed the onset of symptoms. Atrial fibrillation complicates 32% of CTS patients.^4^ Atrial fibrillation is often symptomatic and sometimes causes congestive heart failure in these cases.^4,7^ Catheter ablation is necessary to improve these symptoms, but anatomical complexities in the LA with CTS often make PVI challenging.^8,9^ Atrial fibrillation ablation in congenital heart disease is safe and effective, although the effect depends on the type of AF and/or anatomical complexity.^10^ Although previous reports have mentioned that ICE is helpful to achieve ideal transseptal puncture and precise catheterization, it is difficult to plan safe and effective PVI with ICE, alone.^11–13^ Moreover, age > 80 years is expected to increase the risk of complications with CA, increasing the treatment difficulty. To our knowledge, there is only one published case report of PVI for AF complicated with CTS in an octogenarian.^14^ The report described assessment using an LA model created using a three-dimensional printer, but detailed preoperative anatomical assessment using TTE, TEE, MDCT, and ICE was not mentioned.^14^ In our case, we demonstrated that detailed preoperative anatomical assessment with several imaging modalities led to safe, effective, and successful PVI.

The rate of CA for patients with AF who are older than 80 years of age is increasing owing to increasing healthy life expectancy in ageing societies.^15^ More cases with a clinical course similar to that in our case will reveal the true rate of CTS cases that require PVI. Then, detailed preoperative assessment, which we demonstrated, could contribute to a safe and effective PVI for AF complicated with CTS, even for octogenarians. These preoperative evaluations might be useful to establish the safety and efficacy of PVI in elderly patients with AF not only in CTS cases but also in other congenital heart diseases.

## Data Availability

The data underlying the findings in this article are presented in the article.

## Lead author biography

Dr Yusuke Okuyama graduated from Shiga University of Medical Science in 2009. Currently, he is working as a chief doctor of electrophysiology at the Department of Cardiology in Uji-Tokushukai Medical Center.
